# Simultaneous Degradation of Estrone, 17β-Estradiol and 17α-Ethinyl Estradiol in an Aqueous UV/H_2_O_2_ System

**DOI:** 10.3390/ijerph121012016

**Published:** 2015-09-25

**Authors:** Xiaoyan Ma, Chao Zhang, Jing Deng, Yali Song, Qingsong Li, Yaping Guo, Cong Li

**Affiliations:** 1 College of Civil Engineering and Architecture, Zhejiang University of Technology, Hangzhou 310014, China; E-Mails: mayaner620@163.com (X.M.); 15268157494@sina.cn (C.Z.); seudjing@163.com (J.D.); 2 School of Civil Engineering and Architecture, Zhejiang University of Science and Technology, Hangzhou 310023, China; E-Mail: yali_song@sina.com; 3 Water Resources and Environmental Institute, Xiamen University of Technology, Xiamen 361005, China; E-Mail: Leeqingsong@163.com; 4 College of Biological and Environmental Engineering, Zhejiang University of Technology, Hangzhou 310014, China; 5 College of Civil Engineering and Architecture, Zhejiang University, Hangzhou 310058, China

**Keywords:** UV/H_2_O_2_, steroid estrogens, competitive degradation, photocatalysis, water treatment

## Abstract

UV/H_2_O_2_, which is an advanced treatment technology used to reduce multiple contaminants, is effective in potable water treatment. Simultaneous degradation effects and kinetics of three types of coexisting micropollutant estrogens (steroid estrogens, SEs), including estrone (E1), 17β-estradiol (E2) and 17α-ethinyl estradiol (EE2), in deionized water were studied. Experiments were carried out with ultraviolet-C (UVC) radiation, together with hydrogen peroxide (H_2_O_2_), in a cylinder photoreactor. The results demonstrated that the degradation processes of all of the estrogens strongly fit first-order kinetics. Single solutions of E1, E2 and EE2 showed higher degradation rates and removal efficiencies under the same reaction conditions compared with those under mixed conditions. Coexisting combinations of estrogens were put into the UV/H_2_O_2_ system to estimate their possible competitive influences on each other by examining their removal efficiencies and reaction rate constant, *k*, values. E1 is predominantly reduced rapidly during the competition, while the presence of other estrogens has negligible impacts on E1; however, the degradation of E2 and EE2 is affected by the competitive background, not in relation to the types but to the existing amounts. In the UV/H_2_O_2_ system, photocatalysis of the estrogens can stably produce an intermediate X, with the highest quantity coming from E1, while considerably lower quantities are obtained from E2 and EE2.

## 1. Introduction

Estrogens (steroid estrogens, SEs), including estrone (E1), 17β-estradiol (E2), 17α-ethinyl estradiol (EE2) and estriol (E3), occur in natural environments, such as surface water, seawater and soil, and have attracted significant concern due in part to reports linking these compounds to lower sperm counts in adult males, increases in cancer, and strong and powerful endocrine disrupter activity, even at extremely low concentrations [1,2]. Estrogens have similar tetracyclic structures, with different chemical groups or spatial arrangements at the C-16 and C-17 positions. They exist in the effluent of municipal facilities and some industrial plants, such as those that breed livestock and those that produce prophylactics. These types of pollution are collected during wastewater treatment and cannot be removed efficiently, resulting in their release into natural systems [3,4]. In recent years, the presence of estrogens was reported in both surface water and drinking water in China. Estrogen investigations focused on the Yangtze river, which serves as a water source for many cities, and showed that for the Nanjing section, estradiol equivalent concentrations (EEQcal) reached up to 0.45 ng/L [5]; for the Shanghai section, the maximum concentrations of E2, E1, and EE2 were 0.050, 0.045, and 0.007 ug/L, respectively [6]; for the Jiangsu section, from 2010 to 2012, estradiol equivalent concentrations varied in the range of 0.69–1.15 ng/L [7]. According to a 2007 statistical survey based on an investigation of natural sources in 31 provinces in China, the average value of the equivalent emission of estrogen E2 was 342.0 kg annually, and for the Yangtze River Delta, Jiangsu, Zhejiang and Shanghai, the average values were 238.4, 129.6 and 24.62 kg, respectively [8]. Traditional water treatment used in China cannot effectively remove these compounds; therefore, estrogen pollution was sometimes detected in tap water [9]. Chen [10] verified that chlorine oxidation can only remove 20% to 40% of the estrogen. Through an investigation of the waterworks in south China, Li [11] found that each drinking water treatment process has little effect on the removal of estrogen (approximately 19.5%–50.0%). Because conventional water treatment processes are ineffective in solving the problem, many advanced techniques were investigated, such as ozonation [12,13], activated carbon adsorption and biological degradation. 

The adsorption method can effectively extract trace estrogens from aqueous systems, but pollutants are only transferred from the water phase to the solid phase without decomposition or mineralization [14,15]. The biological degradation of estrogens is more often employed in sewage treatment processes and is not suitable for drinking water treatment due to its slow reduction rates and incomplete decomposition [16,17]. Some oxidants are frequently used in micro-estrogen control, but the effects depend predominantly on the oxidation ability; moreover, incomplete degradation may cause secondary pollution into environment [18,19]. To quickly and efficiently remove trace estrogens from source water with minimum introduction of additional pollutants, the most appropriate methods are combination techniques [20,21]. Presently, research on the control of trace organic pollutants in source water has been given more attention, but more focus has been provided to the analysis of degradation rates, efficiencies and influential factors for a single target pollutant while ignoring simultaneous degradation of pollutants in complex matrices. Based on our former studies, coexisting trace amounts of E1, E2 and EE2 in the UV system can be degraded at different degrees, and the different coexisting combinations of estrogen pollutants used in experiments were designed to summarize simultaneous or probable competitive elimination using UV/H_2_O_2_.

## 2. Materials and Methods

### 2.1. Apparatus

Experiments were performed with a self-designed and self-made photochemical reactor equipped with two 253.7 nm wavelength UV lamps with a total intensity of 350 μW/cm^2^, and the lamps were protected by quartz reaction vessels. The reactor was operated in a recirculation mode by a pump fixed to the outside of the reactor. UV density was confirmed with a photometer. Target pollutants were spiked and mixed to produce a homogeneous distribution. A temperature of 25 ± 1 °C was maintained for reactions. 

### 2.2. Reagents and Instruments

Estrone (standard, ≥99.5%, Dr. Ehrenstorfer GmbH, Augsburg, Germany), 17α-ethinyl estradiol (standard,≥99.3%, Dr. Ehrenstorfer GmbH, Augsburg, Germany), 17β-estradiol (standard, ≥96.8%, Dr. Ehrenstorfer GmbH, Augsburg, Germany), acetonitrile (HPLC grade, 99%, CNW, Shanghai, China), methanol (HPLC grade, 99%, CNW, Shanghai, China), ultrapure water (18 MΩ, Milli-Q, Billerica, MA, USA), deionized water (laboratory-made), and other reagents were of analytical grade. 

A liquid ion trap mass spectrometer (LCQ Decaxp Max, Finnigan, Sunnyvale, Germany), a high performance liquid chromatography system (Agilent1200, Agilent, Santa Clara, CA, USA) with an Eclipse XDB-C18 column (5 μm, 46 × 150 mm, Agilent, Santa Clara, CA, USA), a 12 solid phase extraction device (CNW, Düsseldorf, Germany), a C18 solid phase extraction column (CNW, Düsseldorf, Germany), a 12 hole nitrogen analyzer (OA-SYS Organomation, Berlin, Germany), a UV lamp and quartz tube (253.7 nm, Matt Lighting, Hangzhou, China), and a stainless steel UV reactor (homemade) were used.

### 2.3. Analytical Methods

Residual estrogen concentrations in the aqueous phase were estimated by employing solid phase extraction (SPE). Samples collected at appropriate intervals were filtered through a 0.45 μm membrane and then loaded on a C18 cartridge with 6 cm^3^ and 200 mg packing after the cartridge was conditioned and rinsed with 10 mL methanol and 10 mL ultrapure water sequentially. After sample loading, the cartridges were washed with 10 mL 10% methanol in H_2_O and allowed to dry. Then, 4 mL methanol was used to elute estrogens from the cartridges. Eluents were evaporated to dryness using nitrogen and dissolved in acetonitrile again to a final volume of 1 mL, which was transferred to a vial and analyzed using high performance liquid chromatogram (HPLC). The operating conditions of the chromatographic analysis were set as follows: mobile phase acetonitrile:water = 50:50, flow rate 0.7 mL/min, column temperature 30 °C, detection wavelength 220 nm, and injection sample volume 20 μL. The analyte retention times were 5.0, 6.2, and 7.2 min for E2, EE2, and E1, respectively. 

Samples for byproduct identification were collected from the UV/H_2_O_2_ system with a photo intensity of 350 µW/cm^2^ and 10 mg/L hydrogen peroxide (H_2_O_2_), and the samples were pretreated by SPE as described above. Mass spectrometry conditions were set at a sheath gas flow rate of 20 a.u (Arb. units), spray voltage of 3 kV, and capillary temperature of 300 °C in full scan mode.

### 2.4. Batch Experiments

Competitive degradation experiments for tri-coexisting model analysis were carried out by adding three types of estrogens to ultrapure water and then diluting the solutions with deionized water to achieve the desired concentration for each target (50 μg/L). The photo intensity was set at 350 μW/cm^2^, and the H_2_O_2_ dose was altered to 5, 10 and 15 mg/L, sequentially. 

Competitive degradation experiments for dual-coexisting model analysis were carried out by adding two types of estrogens, either E2 and EE2, E1 and EE2, or E1 and E2, to ultrapure water and then diluting the solutions using deionized water to achieve the desired concentration for each target (50 μg/L). The photo intensity was set at 350 μW/cm^2^, and the H_2_O_2_ dose was set at 10 mg/L. Single estrogen degradation experiments were carried out for comparison with the dual-coexisting model. 

For each experiment, samples that were collected at appropriate time intervals (0, 2, 5, 10, 15, 30, 50, 80, and 120 min) were enriched by solid phase extraction and analyzed by HPLC.

## 3. Results and Discussion

### 3.1. Simultaneous Degradation of E1, E2 and EE2 in UV/H_2_O_2_ System

**Figure 1:**
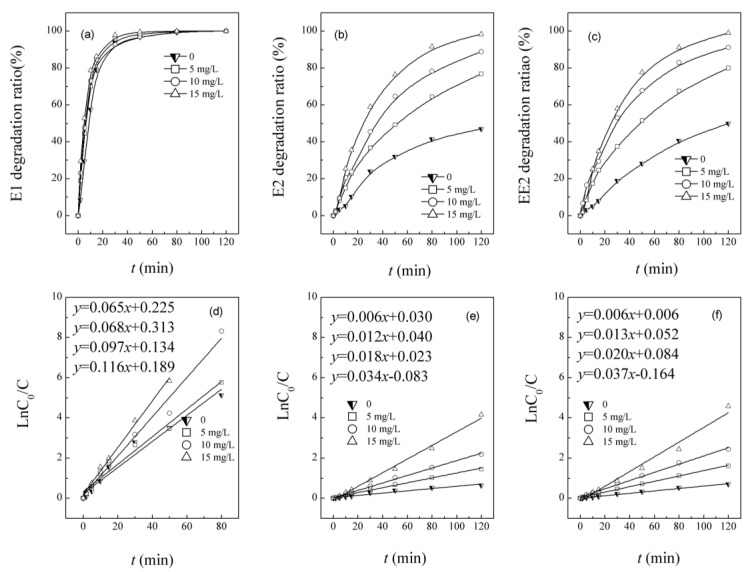
Degradation kinetics of mixed pollution estrone (E1), 17β-estradiol (E2) and 17α-ethinyl estradiol (EE2) under UVC and UV/H_2_O_2_.

Figure 1 shows the simultaneous photolysis and photocatalysis of mixed E1, E2 and EE2 in the UV/H_2_O_2_ system with H_2_O_2_ doses of 0, 5, 10 and 15 mg/L.

Under UVC radiation with or without hydrogen peroxide (H_2_O_2)_, the degradation of estrogens follows apparent first-order kinetics. When H_2_O_2_ was absent, estrogens were photolyzed by UVC. The degradation sequence observed in Figure 1 follows E1 > E2 ≈ EE2, which is similar to the conclusions of Liu [22] and to those of our former studies [23]. Single UVC showed fast emulation of E1 with a conversion of 97.0% after 50 min of radiation; however, only 32.0% and 28.2% of E2 and EE2 was observed, respectively. The faster degradation of E1 can be explained by its much stronger absorption of photons in the ultraviolet-C (UVC) region of the electromagnetic spectrum. Estrogens have photosensitive phenolic structures [24] and can be efficiently reduced by photolysis. Liu [22] notes that UVC can rapidly degrade E1 by direct photolysis, in which the pollutant molecules absorb photon energy, transit to a high level, and then undergo irreversible reactions that result in molecular bond fractures, *etc.* In his paper, UVA was also found to be efficient in E1 reduction but had no effect on E2, which suggested that the breakdown energy of the chemical bond in E2 is higher than that of E1. Whidbey [25] also discovered that direct photodegradation of E1 was faster than that of E2 and EE2, with a shorter half-life.

UV/H_2_O_2_ can significantly improve the conversion of estrogens, and mixed estrogens follow the degradation sequence E1 > EE2 > E2. The result was similar to Puma’s [25] conclusion. Under direct radiation of UVA and UVC, the conversion sequence was also E1 > EE2 > E2. The UV absorbance at 254 nm showed that E1 had the greatest absorbance value of 0.042 cm^−1^, more than 0.021 and 0.026 cm^−1^ of E2 and EE2, respectively. This proves that E1 has much stronger photon absorption capability. H_2_O_2_ was added to enhance the efficiencies of conversion. With an H_2_O_2_ dose of 5 mg/L, the degradation of E1 shows a slight enhancement of 97.0% conversion, while the removal of E2 and EE2 was increased much more, reaching 49.2% and 51.7%, respectively, after 50 min of contact time. When the H_2_O_2_ dose was increased to 15 mg/L, the removal efficiencies of E1, E2 and EE2 increased to 99.7%, 76.4% and 77.6%, respectively, which was more effective than single UVC radiation; H_2_O_2_ in solution acts as electron acceptor, absorbs photons under ultraviolet radiation, and produces free hydroxyl radicals as a result of O–O bond ruptures. Free hydroxyl radicals can subsequently react with estrogens [6]. With increased H_2_O_2_ doses, an increased number of free hydroxyl radicals are produced, and hence, the removal efficiencies of E1, E2 and EE2 are increased. The results suggested that hydroxyl radicals formed at 10 mg/L H_2_O_2_ produce almost equivalent reductions of E2 and EE2 with photolysis; therefore, the 10 mg/L H_2_O_2_ dose was selected in the following experiments to provide further understanding of simultaneous estrogen removal in the UV/H_2_O_2_ system to see if there are competitive effects within various coexisting combinations of E1, E2 and EE2. 

### 3.2. Competitive Influence of E2 and EE2 on E1 Degradation

**Figure 2:**
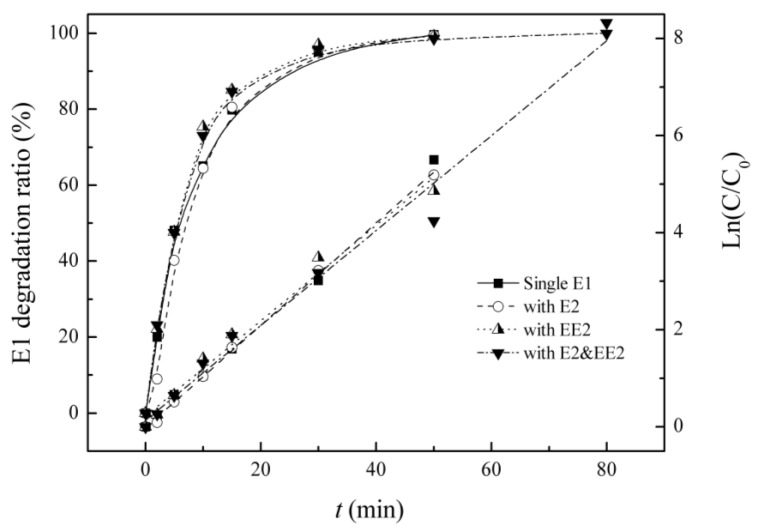
Degradation and first order kinetics of E1 under single and combined estrogen pollution.

Single E1 pollution, mixed solutions of EE2 and E1 (dual-solution) and of E1 and E2, and a solution containing E1, E2 and EE2 (tri-solution) were prepared in the reactor. Residual E1 was measured, and the influence of coexisting SEs is shown in the Figure 2 and Table 1.

**Table 1:** Kinetic parameters of degradation of E1 under single and combined estrogen pollution.

Background Estrogens	Kinetic Equation	Reaction Rate Constant K/(min^−1^)	R^2^	Half-lIfe/(min)
Blank	Ln(C_0_/C) = 0.107*t* + 0.001	0.107	0.996	6.4
E2	Ln(C_0_/C) = 0.106*t −* 0.015	0.106	0.999	6.5
EE2	Ln(C_0_/C) = 0.098*t* + 0.218	0.098	0.979	7.0
E2&EE2	Ln(C_0_/C) = 0.098*t* + 0.134	0.098	0.981	7.1

Influences from co-solute estrogens, such as E2 or EE2, show inhibitory effects on E1 degradation. There is little difference between various backgrounds. E2 and EE2 showed almost the same degree of influence on E1, maybe because the two compounds have similar absorbance character. Mazellier [24] had reported that E2 and EE2 had very similar quantum yield upon irradiation at 254 nm, and thus the two compounds had similar photolysis efficiencies. After 50 min reactions, the removal percentages of E1 are 99.6%, 99.4% and 99.2% in single solutions and in dual solutions of E2 and E1 and of EE2 and E1, respectively, and the removal efficiency decreases to 98.6% in tri-solution. Under simulated combination pollutant conditions, the degradation of E1 follows pseudo-first-order reaction kinetics, and the reaction rate constants and half-life reveal a slight decline in E1 degradation with increasing co-pollutant concentration in the UV/H_2_O_2_ system. The presence of either E2 or EE2 has a competitive effect on the degradation of E1, but the impact is slight, possibly because E1 has the strongest photosensitivity and a smaller requirement for hydroxyl radicals (•OH).

### 3.3. Competitive Influence of E1 and EE2 on E2 Degradation

**Figure 3:**
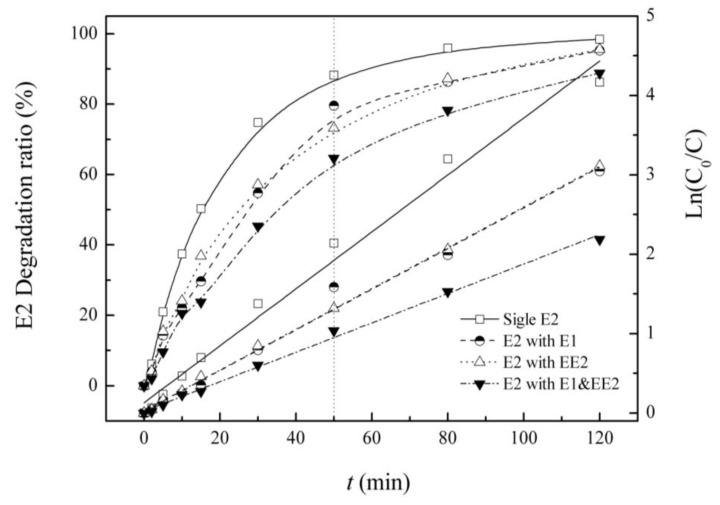
Degradation and reaction kinetics of E2 under single and combined estrogen pollution.

E2 alone, mixed solutions of E2 and E1 and of E2 and EE2, and the tri-solution E1, E2 and EE2 were prepared in the reactor. Residual E2 was measured, and the influence of coexisting steroid estrogens (SEs) are shown in Figure 3 and Table 2.

**Table 2:** Kinetic parameters of degradation of E2 under single and combined estrogen pollution.

Background Estrogens	Kinetic Equation	Reaction Rate Constant K/(min^−1^)	R^2^	Half-Life/(min)
E2	Ln(C_0_/C) = 0.036t + 0.129	0.036	0.985	19.3
E1	Ln(C_0_/C) = 0.026t + 0.025	0.026	0.988	27.0
EE2	Ln(C_0_/C) = 0.026t + 0.029	0.026	0.999	26.9
E1&EE2	Ln(C_0_/C) =0.018t + 0.023	0.018	0.996	37.4

From the degradation curves, it can be concluded that E2 degradation is significantly affected by the background of other estrogens. The co-solute estrogens inhibit E2 removal. On the one hand, with the extension of the reaction time, the inhibition effect first increases and then decreases. As the reaction time is extended to 120 min, the E2 removal ratio is 98.5%, and although the removal efficiencies in the dual-solutions with E1 and EE2 are reduced (95.5% and 95.2%), these are still minor differences *versus* single E2; under tri-solute conditions, the E2 removal rate is only 88.8%, which is far below that of the single E2. On the other hand, as the types of co-existing estrogens increase, inhibition gradients show rising trends. In the range of 15–80 min, the difference in the gradients is significantly high, and E2 removal ratio as a single pollutant can reach 50.3%–95.9%; for E1 as the background estrogen, the removal ratio is 29.65%–86.3%; for EE2 as the background estrogen, the removal ratio is 36.95%–87.2%; and for E1 and EE2 as a combined background, the removal ratio is 23.85%–78.3%.

Under different conditions of estrogen pollution, the degradation of E2 in the UV/H_2_O_2_ process follows the pseudo-first-order reaction kinetics model. Reaction rate constants and half-lives of E2 reveal competition with other estrogens. The E2 single degradation rate constant was 0.036 min^−1^, while when E1 and EE2 were the background estrogens, the value decreased to 0.02568 min^−1^, which is almost the same. The equivalent effect of E1 or EE2 on E2 is accounted for by the competition for hydroxyl radicals (•OH). When three types of estrogen coexist, the lowest rate constant of 0.1853 min^−1^ also suggests this reason.

### 3.4. Competitive Influence of E1 and E2 on EE2 Degradation

**Figure 4:**
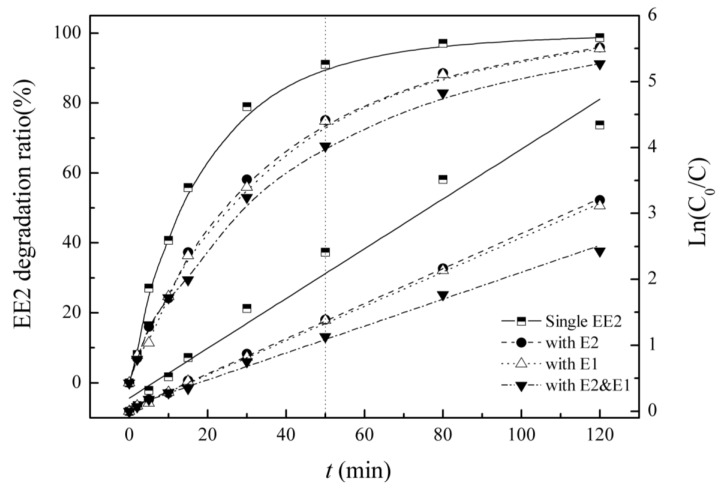
Degradation and reaction kinetics for EE2 under single and combined estrogen pollution.

EE2 alone, mixed solutions of EE2 and E1 and of E2 and E2, and the tri-solution of E1, E2 and EE2 were prepared in the reactor. Residual EE2 was measured, and the influence of coexisting SEs is shown in Figure 4 and Table 3.

**Table 3:** Kinetic parameters of degradation of EE2 under single and combined estrogen pollution.

Background Sterdidal Estrogens	Kinetic Equation	Reaction Rate Constant K/(min^−1^)	R^2^	Half-Life/(min)
EE2	Ln(C_0_/C) = 0.038t+ 0.199	0.038	0.973	18.4
E2	Ln(C_0_/C) = 0.026t + 0.037	0.026	0.999	26.1
E1	Ln(C_0_/C) = 0.026t + 0.029	0.026	0.999	26.6
E2 & E1	Ln(C_0_/C) = 0.020t + 0.067	0.020	0.995	33.9

EE2 degradation curves under different simulated conditions have similar characteristics to those observed for E2. E1 and E2 have inhibitory effects on the degradation of EE2. When reaction time was extended, the inhibitory effects were first reinforced and then decreased; when the estrogen type was increased, the inhibitory effects were strengthened. 

Dual-solution experiments show that E1 exerts almost the equivalent inhibition on EE2 to that of E2. 

EE2 degradation processes in the UV/H_2_O_2_ system are consistent with the first order kinetic model under different combination conditions, and the reaction rate constant and the half-life show influences of co-existent estrogens on EE2. 

### 3.5. Intermediate Product of Estrogens in UV/H_2_O_2_ System

**Figure 5:**
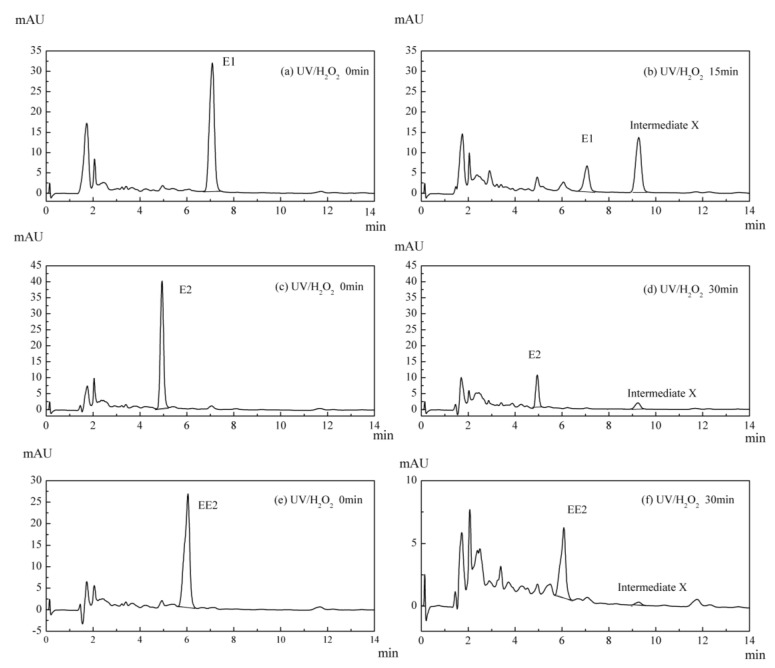
HPLC chromatogram of estrogens and the intermediate product X in UV/H_2_O_2_ system.

In the UV/H_2_O_2_ system, liquid chromatography was used to detect residual E1, E2 and EE2. The chromatogram showed that a stable peak of an intermediate product at a retention time of 9.24 min appeared for each estrogen. The initial chromatograms of the original substances are compared with those of the 15 or 30 min reactions, and the degradation intermediate X is shown in Figure 5. 

**Figure 6:**
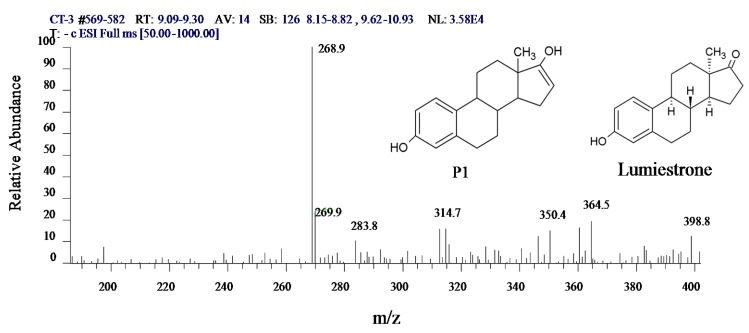
Mass chromatogram and the probable structure of intermediate X.

The characteristic ions of E1 are 268.8, 268.8, 268.8 and 338.9, while the ions for intermediate X are 268.9, 269.9, 364.5 and 314.7. Steroid estrogens are categorized as strong aromatic compounds due to the large energy consumption of their conjugated bonds, and the most frequent peak of the mass spectrogram is 268.9. Intermediate X has a similar chemical structure to that of E1 based on a comparison of the peak and its characteristic ions. Caupos *et al.* [26] reported that without dissolved organic carbon (DOC) in the water, E1 degradation by ultraviolet can generate one unique product, which is designated as P1, and it is held longer than E1 in HPLC analysis. Contractive analysis using gas chromatogram-mass spectrum (GC-MS), liquid chromatogram-mass spectrum (LC-MS), liquid chromatogram-mass spectrum-mass spectrum (LC-MS2), and LC-MS3 revealed that P1 is likely to be an isomer of E1, and the predicted structure is shown in Figure 6. Trudeau *et al.* [27] found that under the irradiation of UV-B, E1 generates a photodegradation derivative, which was identified as 13α-epimer lumi-estrone (lumiestrone) by Nuclear magnetic resonance (NMR) analysis, as shown in Figure 6. Whidbey *et al.* [28] subsequently studied the conversion rate of E1 to LumiE1 based on the former results and concluded that LumiE1 had less estrogen effects than E1, partly because the structure change from E1 to LumiE1 was mostly a methyl transformation.

### 3.6. Variation in the Characteristics of Intermediate X in the UV/H_2_O_2_ System

**Figure 7:**
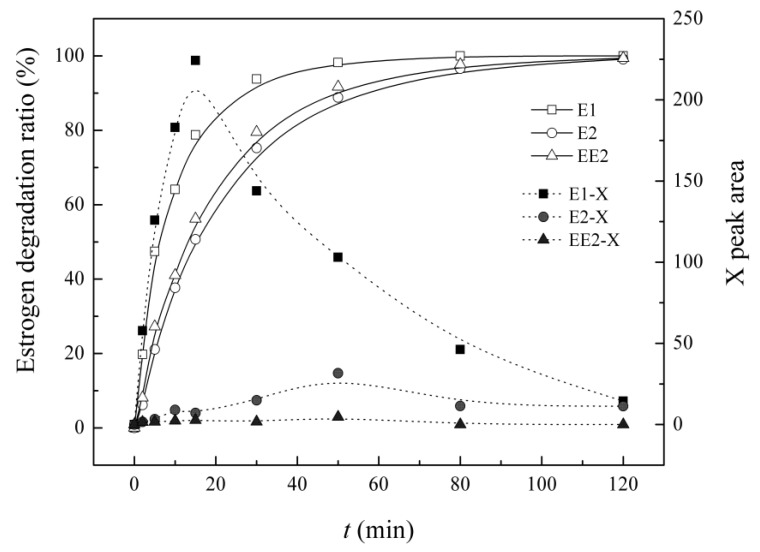
Variation of intermediate X along with single estrogen degradation in UV/H_2_O_2_ system.

When estrogens were separately degraded by UV/H_2_O_2_, intermediate X always showed at retention time (RT)= 9.24 min. Its varying characteristic curves, along with estrogen degradation, are shown in Figure 7. At equivalent initial concentration levels, the greatest yield of X was generated from E1, and it was tremendously decreased for E2 and EE2, particularly EE2. Along with the degradation of the target estrogens, the level of intermediate X first increases rapidly to the maximum value and then decreases gradually. The maximum production of E1 appeared at 15 min, while for E2 and EE2, it was delayed to 30 min. X gradually decreased, mainly due to the redox reaction in the UV/H_2_O_2_ system. 

**Figure 8:**
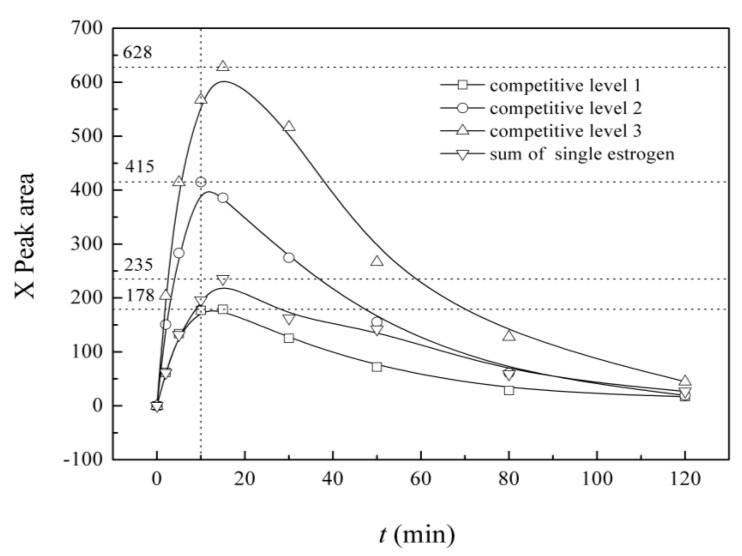
Variation of intermediate X under different simulated conditions in UV/H_2_O_2_ system (competitive level 1: initial cosolute concentration C_0_ of E1 = 45.2 μg/L, E2 = 43.2 μg/L and EE2 = 44.4 μg/L; initial cosolute concentration C_0_ of level 2: E1 = 86.3 μg/L, E2 = 87.0 μg/L and EE2 = 83.3 μg/L; initial cosolute concentration C_0_ of level 3: E1 = 135.3 μg/L, E2 = 140.9 μg/L and EE2 = 127.8 μg/L; initial single estrogen concentration C’_0_ of E1 = 47.2 μg/L, E2 = 44.3 μg/L and EE2 = 41.0 μg/L).

X variation curves for estrogens experiencing competitive degradation at different initial concentrations (approximately 400, 800 and 1200 μg/L, where the measured concentrations are addressed under the figure) are shown in Figure 8. Quantities of X produced from three types of estrogens under single estrogen degradation conditions were recalculated based on the initial concentrations of the simultaneous situation, both separately and added together. Under the same initial concentrations, the amount of X that was generated in the competition situation is lower than the single estrogen degradation condition because of the mutual inhibitory effect. In competitive models, along with an increase in the initial estrogen concentration, the amount of X gradually increases, and the highest amount appeared almost at the same time, which was approximately 15 min, while after that, the level of X began to decrease.

## 4. Conclusions

This work has shown that μg/L concentrations of estrogens can be simultaneously degraded by advanced oxidation using UVC plus a certain dose of H_2_O_2_. E1 can be effectively removed by UVC and UV/H_2_O_2_ within 50 min because direct radiation plays a major role, while it takes 120 min to remove E2 and EE2 effectively. For coexisting estrogens, the degradation sequence follows E1 > EE2 > E2 in the UV/H_2_O_2_ system. The coexistence of separate E2 and EE2 with E1 has little competitive effect on E1 degradation, while E1 and E2 (or EE2) have significant and equal inhibition effects on coexisting EE2 (or E2) removal. In the UV/H_2_O_2_ system, estrogens of E1, E2 and EE2 can produce the same intermediate product, which has a similar chemical structure to that of E1.

## Abbreviations

SEs: steroid estrogens
E1: estrone
E2: 17β-estradiol
EE2: 17α-ethinyl estradiol
SPE: solid phase extraction
